# Propolis envelope in *Apis mellifera* colonies supports honey bees against the pathogen, *Paenibacillus larvae*

**DOI:** 10.1038/s41598-017-11689-w

**Published:** 2017-09-12

**Authors:** Renata S. Borba, Marla Spivak

**Affiliations:** Department of Entomology, University of Minnesota, 1980 Folwell Ave, Saint Paul, MN 55108 USA

## Abstract

Honey bees have immune defenses both as individuals and as a colony (e.g., individual and social immunity). One form of honey bee social immunity is the collection of antimicrobial plant resins and the deposition of the resins as a propolis envelope within the nest. In this study, we tested the effects of the propolis envelope as a natural defense against *Paenibacillus larvae*, the causative agent of American foulbrood (AFB) disease. Using colonies with and without a propolis envelope, we quantified: 1) the antimicrobial activity of larval food fed to 1–2 day old larvae; and 2) clinical signs of AFB. Our results show that the antimicrobial activity of larval food was significantly higher when challenged colonies had a propolis envelope compared to colonies without the envelope. In addition, colonies with a propolis envelope had significantly reduced levels of AFB clinical signs two months following challenge. Our results indicate that the propolis envelope serves as an antimicrobial layer around the colony that helps protect the brood from bacterial pathogen infection, resulting in a lower colony-level infection load.

## Introduction

The nests of densely populated social insect colonies provide a favorable habitat for a wide range of parasites and pathogens that have evolved to overwhelm or suppress their hosts’ immune defenses^1^. In turn, insect societies have evolved remarkable abilities to counter these challenges via dynamic defense mechanisms at both the individual level (individual immunity^2^; e.g., cell phagocytosis, immune gene up-regulation) and the colony level (social immunity^3^).

An example of social immunity is the collection of antimicrobial plant resins^4–12^. Social wood ants (*Formica paralugubris*) collect plant resins and place globules of resin near the brood^9^, resulting in reduced growth of microorganisms^10^, and lower immune system activity of adult worker ants^11^. Honey bees, *Apis mellifera*, collect plant resins and deposit these resins on the interior walls of the nest, where it is called a propolis envelope^4, 7^. Previous work has shown that the presence of a propolis envelope within the nest provides benefits to adult bees’ by lowering constitutive immune gene expression^6, 8^. The immune system may be the most energetically costly physiological system in insects^1, 13^. Thus, social immune behaviors, such as the collection of plant resins, may have evolved to reduce the need to maintain a highly activated immune system (constitutive immune defense) when the insect is not pathogen challenged. Additionally, the antimicrobial properties of plant resins may also control pathogen loads and promote colony health^5, 6, 10, 12^.

*In vitro* studies have demonstrated that propolis, and specific compounds within propolis, inhibit the growth of two infectious pathogens of honey bees, *Paenibacillus larvae* and *Ascosphaera apis* ^14–19^. When colonies in the field were challenged with *P*. *larvae* and subsequently treated with propolis *per os* via an aqueous, alcohol or sugar solution, the treatment reduced spore loads and disease signs from the hive^16, 20^. Simone-Finstrom and Spivak^5^ found that colonies with an experimentally applied propolis envelope and challenged with *A*. *apis* had significantly lower clinical signs of the disease. These results suggest that the antimicrobial properties of propolis may aid in reducing pathogen loads, but do not completely kill or render inactive the disease agents.

American foulbrood (AFB), one of the most infectious bacterial diseases of honey bees, is caused by *P*. *larvae*. Although adult bees may come in contact with *P*. *larvae* spores and become carriers, only young bee larvae (1–2 d old) are susceptible to this pathogen. One route of AFB transmission to young larvae is via oral intake of contaminated larval food supplied by nurse bees^21^. Young larvae are thought to have lower immune defenses compared to adults as indicated by lower hemolymph cell counts and phenoloxidase activity^22, 23^. Therefore, young larvae may rely mostly on adult bee social immunity to avoid contracting and to fight infectious brood diseases^24–28^. For example, nurse bees secrete antimicrobial compounds into larval food, which help protect the larvae from *P*. *larvae* infection^29–31^. Adult bees (e.g., nurse bees) display increased transcription of antimicrobial peptides (immune-related genes) 24 h after challenge with *P*. *larvae*, even though they are only carriers of the pathogen^2^.

In this study, we challenged colonies with *P*. *larvae* and investigated the effects of the propolis envelope on: 1) the antimicrobial activity of larval food fed to 1–2 d old larvae; and 2) the overall reduction of clinical signs of AFB in the colony. We hypothesized that antimicrobial activity of larval food would be higher, and clinical signs of AFB would be reduced in *P*. *larvae* challenged colonies with a propolis envelope compared to challenged colonies without the envelope. Our findings supported our hypotheses and emphasize the critical importance of the propolis envelope to honey bee health and its role in larval defense against bacterial infections.

## Results

### Larval food antimicrobial activity

**Figure 1:**
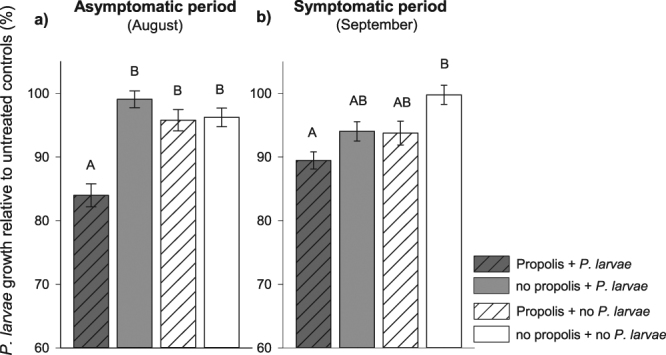
Larval food inhibitory activity against *Paenibacillus larvae* growth in colonies with and without a propolis envelope, and challenged or not with *Paenibacillus larvae*. Antimicrobial activity of larval food (mean ± s.e.m.) for samples collected in: (**a**) Asymptomatic period in August, and (**b**) Symptomatic period in September were measured as percent optical density (OD_600_) relative to untreated controls (N = 20 replicate wells per colony and five colonies per treatment). Low *P*. *larvae* growth (y-axis) indicates higher inhibition activity of larval food. Significant difference among groups was determined by ANOVA with colony treated as a random variable (α = 0.05).

Larval food from 1–2 d old larvae from colonies with and without a propolis envelope showed differences in the ability to inhibit the growth of *P*. *larvae* both before and after the appearance of AFB clinical signs in the colonies. Asymptomatic period (August): Larval food collected from challenged colonies with a propolis envelope showed significantly higher inhibition of *P*. *larvae* growth (lower growth relative to controls) compared to larval food from all other three treatment groups (*F*_3,16_ = 5.16, *P* = 0.011; Fig. 1a). Symptomatic period (September): When challenged colonies had clinical signs of AFB, larval food collected from challenged colonies with a propolis envelope continued to show significantly higher inhibitory activity against *P*. *larvae* but only compared to larval food from unchallenged colonies without a propolis envelope (*F*_3,16_ = 3.27, *P* = 0.049; Fig. 1b). Larval food from unchallenged colonies with a propolis envelope and challenged colonies without a propolis envelope had intermediate levels of inhibition.

### Level of American foulbrood infection

**Figure 2:**
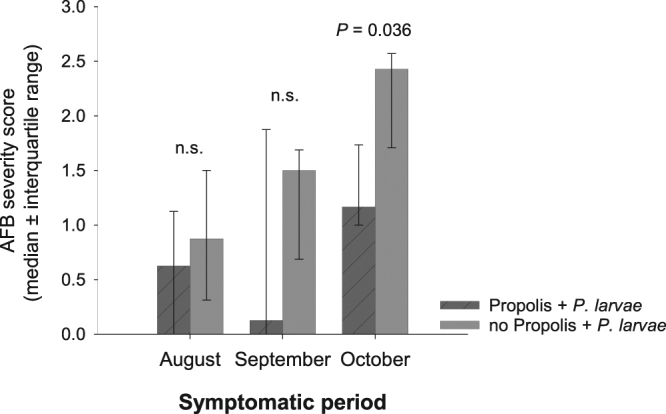
American foulbrood infection level in challenged colonies with and without a propolis envelope. Overall severity scores (0 = 0 cells in comb containing sign of AFB; 1 = 1–5 cells; 2 = 6–25 cells; and 3 =≥ 26 cells per comb) were compared between challenged colonies with and without a propolis envelope using Mann-Whitney U test (α = 0.05). Three out of five challenged colonies with a propolis envelope and four out of five challenged colonies without a propolis envelope showed signs of AFB infection in both August and September. High interquartile range in September in challenged colonies with a propolis envelope was a result of one colony showing a high AFB infection score level compared to all others. All colonies, in both challenged treatment groups, showed signs of AFB infection in October.

Colonies first showed clinical signs of AFB 30 days after challenge. On August 30^th^, *P*. *larvae* challenged colonies with a propolis envelope had low but comparable numbers of larvae with clinical signs of AFB (median score of 0.625) compared to challenged colonies without a propolis envelope (median score of 0.875; *U* = 16, *P* = 0.523). Similar results were observed on September 16^th^ (*U* = 16.5, *P* = 0.456). Three out of five challenged colonies with a propolis envelope and four out of five challenged colonies without a propolis envelope showed signs of AFB infection in both August and September. This difference was not statistically significant until October 1^st^, when colonies with a propolis envelope had significantly fewer larvae with clinical signs of AFB (*U* = 23, *P* = 0.036; Fig. 2).

The presence of the propolis envelope did not completely prevent AFB infection; all challenged colonies with a propolis envelope had clinical signs of infection by the end of the experiment. However, the severity of clinical signs in October in colonies with a propolis envelope was relatively mild (median score of 1.17, equivalent to 1-5 infected larvae per comb) compared to the severity in colonies without the propolis envelope (median score of 2.43, equivalent to 6–25 infected larvae per comb).

## Discussion

The presence of a propolis envelope in healthy managed honey bee colonies provides quantifiable benefits to honey bee immunity^6, 8^. In this study, we demonstrate two benefits of the propolis envelope to bees after field colonies were challenged with *P*. *larvae*, the causative agent of American foulbrood (AFB) disease. First, before the colonies were symptomatic, larval food from challenged colonies with a propolis envelope had significantly higher inhibitory activity against *P*. *larvae* compared to larval food from challenged colonies without a propolis envelope. Second, the presence of a propolis envelope inside a colony resulted in a significant reduction in the number of larvae with clinical signs of AFB two months after *P*. *larvae* challenge.

*Paenibacillus larvae* can cause lethal infection only to young larvae. Although *P*. *larvae* spores do not germinate in adult bees, these bees play an important role in disease spread within the colony. One of the earliest studied mechanisms of AFB resistance was associated with anti-foulbrood compounds found in larval food^29, 30^. Data from our preliminary studies indicated that after challenge with *P*. *larvae*, nurse-age bees in challenged colonies with a propolis envelope were able to mount a significantly higher immune response compared to same-age bees in challenged colonies without a propolis envelope^32^. Although our data require confirmation, they suggest one possible mechanism for the increase in antimicrobial activity of the larval food. In the presence of AFB infection, nurse bees in colonies with a propolis envelope may be able to induce a stronger up-regulation of antimicrobial peptides and incorporate these peptides into larval food. The presence of antimicrobial peptides in larval food may protect them from *P*. *larvae* infection^29–31^, and decrease colony-level AFB infection more rapidly and efficiently compared to bees from challenged colonies without a propolis envelope. The transfer of antimicrobial compounds as a proposed bacterial defense has been observed in another social insect, carpenter ants (*Camponotus pennsylvanicus*). Infected worker ants demonstrate increased trophallaxis to naïve nestmates. During trophallaxis, infected workers share droplets of antimicrobial peptides with naïve individuals, improving the survival of naïve individuals upon future contact with the same bacterial pathogen^33^.

There may be a second possible mechanism for the increase in antimicrobial activity of the larval food. Although the propolis envelope may not come into direct contact with larval food, volatile compounds present in propolis can diffuse through the hive, and possibly into the larval food, contributing to the complex way in which bees fight infections. Several studies have confirmed the activity of propolis volatiles against Gram-positive^15, 34–36^, and Gram-negative bacteria^35–37^, as well as the inhibitory activity of non-volatile compounds against *P*. *larvae* ^19^. Propolis is a mixture of resins, which may contain different active compounds against *P*. *larvae*. For examlpe, the most active constituents of Bulgarian propolis against *P*. *larvae* growth, comprised of resin from *Populus* spp, were pinocebrin, pinobanksin-3-acetate, and a caffeate mixture^15^. More recently, six 3-acyl dihydroflavanols compounds, active against *P*. *larvae* growth, were isolated from U.S. propolis^19^. All of the active dihydroflavanols were present, in varied concentrations, in several North American *Populus spp*., including *P*. *deltoides*, the most abundant resin source in our research apiary area on the St. Paul campus of the University of Minnesota. It has not been investigated if propolis compounds are present in larval food, although it has been suggested that antimicrobial compounds found in propolis are present in honey^38^.

The relatively high bioactivity of the larval food observed only in samples from challenged colonies with a propolis envelope suggests there may be synergistic or additive effects between active constituents in propolis and antimicrobial substances present or incorporated into larval food by adult bees. A synergism between propolis and antimicrobial compounds, including antibacterial compounds, has been previously reported to augment the effect of commonly used drugs to combat human diseases^39, 40^. The source of the growth-inhibiting substances put in larval food by nurse bees, from glandular secretions (e.g., antimicrobial peptides;^31–33, 41^) or midgut contents (e.g., lactic acid bacteria^42, 43^), requires further study. In sum, the consistently higher antimicrobial activity of honey bee larval food in challenged colonies with propolis reveals an inhibitory effect of the propolis envelope against *P*. *larvae* and a protective physiological response of nurse bees towards the brood.

In regards to the effect of propolis on AFB infection level, our results indicate that the presence of a propolis envelope inside a colony significantly reduced the number of larvae with clinical signs of AFB over time, but did not eliminate the disease completely. Colonies challenged with *P*. *larvae*, with and without a propolis envelope, had similar levels of AFB infection in late August and mid-September. By early October when queens were still actively laying eggs, infection levels in colonies with a propolis envelope were significantly lower compared to levels in colonies without a propolis envelope.

Previous studies have demonstrated several mechanisms of colony resistance to AFB, including: removal of *P*. *larvae* spores from contaminated honey by action of the proventricular valve in the honey bee crop^26^; detection and removal of infected larvae by adult bees before *P*. *larvae* produces infectious spores (hygienic behavior^25^); genetic resistance in larvae^27, 28^; and high inhibitory activity of larval food on the growth of *P*. *larvae* ^29^. Here we demonstrate an additional, and novel, mechanism of colony resistance associated with the presence of a propolis envelope within the honey bee colony. Specifically, we demonstrate an increased bioactivity of larval food against *P*. *larvae* growth when honey bees inhabit colonies containing a propolis envelope.

Managing honey bees in man-made hives with smooth interior walls has interfered with a critical, natural defense mechanisms of the honey bee colony: the bees do not construct a natural propolis envelope inside the hive as they do in natural tree cavities. Our results provide additional evidence for the importance of the propolis envelope within the nest, in this case protecting the brood from *P*. *larvae* infection, resulting in a lower infection load two months following the challenge. The propolis envelope can be viewed as an external component of the bees’ immune defense mechanism and thus as a vital part of honey bee colony defense.

## Materials and Methods

### Experimental design

Twenty, four-frame “nucleus” colonies were purchased in May 2013 and established in 10-frame equipment at the University of Minnesota, Saint Paul, MN, U.S.A. Hygienic tests were performed on all colonies to ensure the colonies did not display hygienic behavior^44^, a behavioral mechanism of resistance to AFB in which the bees detect and remove immature bees infected with *P*. *larvae* ^25^. Ten colonies were provided with commercially available propolis traps (Mann lake LTD, Minnesota) stapled to the four inner walls of each bee box to encourage the bees to construct a propolis envelope within the nest, following previous methods^8^. Colonies that were provided with propolis traps deposited propolis in more than 80% of the slits in the traps, creating a propolis envelope (Supplementary Fig. S1). In total, four treatments were tested in this study. Five of the ten colonies with a propolis envelope were challenged with *P*. *larvae* (propolis + *P*. *larvae* − Treatment 1) and the other five colonies were left unchallenged (propolis + no *P*. *larvae* − Treatment 2). The remaining ten colonies were not provided with propolis traps and the bees deposited small, scattered amounts of propolis in the cracks and crevices within the boxes. Five of the ten colonies without a propolis envelope were challenged with *P*. *larvae* (no propolis + *P*. *larvae* − Treatment 3) and the other five were left unchallenged (no propolis + no *P*. *larvae* − Treatment 4).

### Colony inoculation with *P*. *larvae*

Sugar solution containing 10^7^ *P*. *larvae* spores/ml as prepared by removing 100 desiccated larvae that died from AFB infection (i.e., AFB “scales”) from diseased colonies, and macerating and suspending the crushed scales in sucrose-water (1:10 w/v)^45^. Spore concentration was confirmed using a haemocytometer. Colonies were challenged with *P*. *larvae* on July 31^st^ 2013 by spraying 5 ml of the spore solution on each comb within the colony^46^. Unchallenged colonies were sprayed with 5 ml of sugar solution (1:10 w/v) on each comb within the colony.

### Larval food collection

Prior to larval food collection, an empty frame was introduced into the colony and marked when eggs were present. Three days after the frames were marked, when 1–2 d old larvae were present, the frames were removed and larval food was collected following Schmitzová *et al*.^47^. Larval food from cells containing 1–2 d old larvae was collected 9 days after colony inoculation with *P*. *larvae* (asymptomatic period, August 9^th^) and after the presence of clinical signs (symptomatic period, September 12^th^, 43 days after challenge). Larval food from 20 cells, located in the same frame, was collected from each colony and stored individually. In a temperature-controlled room, each young larva was removed from the cell using a sterile grafting tool, the larval food from each cell was individually homogenized in 30 μl of phosphate buffer (50 mM NaH_2_PO_4_/Na_2_HPO_4_, pH 7.0; 100 mM NaCl; 20 mM EDTA, pH 8.0) by repeated pipetting and then transferred to a 1.5 ml microcentrifuge tube. An average of 2 μl of larval food was collected from each larval cell.

### Larval food antimicrobial assay

Prior to the assay, *P*. *larvae* (from stock strains obtained from the USDA Agricultural Research Service culture collection; NRRL# B-2605) were cultured in brain/heart infusion broth for 48 h. A bacterial growth assay was performed to assess the inhibitory activity of larval food collected from cells containing 1–2 d old larvae against the growth of *P*. *larvae*. The bacterial growth assay was conducted following Wilson *et al*.^18^ with minor modifications. Larval food in phosphate buffer and controls (30 μl of phosphate buffer) were freeze-dried and solubilized in 100 μl of brain/heart infusion broth, transferred to 96-well plates and placed in a plate shaker for 30 min at 400 rpm to improve homogenization. A volume of 100 μl of *P*. *larvae* liquid culture (cultured for 48 h prior to the assay) was transferred into the well plates, and the well plates were incubated at 37 °C at 400 rpm for six hours. Bacterial growth inhibition was evaluated in 96-well plates by measuring turbidity of treated cultures (containing larval food) relative to untreated controls (phosphate buffer only) using a microplate spectrophotometer. Turbidity was measured as the optical density at time 0 h subtracted from the optical density at time 6 h (OD_600_).

### Level of American foulbrood (AFB) infection assessment

The number of larvae with clinical signs of AFB (sunken wax capping and uncapped cells containing discolored, “ropy” brood) was quantified every 16 days ( ± 1 day) after the appearance of the first clinical signs (i.e., August 30, September 16 and October 1, 2013). A severity score ranging from 0–3 was given for each comb (both sides combined) that contained larvae: 0 = 0 cells containing signs of AFB; 1 = 1–5 cells; 2 = 6–25 cells; and 3 =≥ 26 cells per comb^25^.

### Statistical analysis

*P*. *larvae* growth was calculated by subtracting the optical density (OD) at time 0 h from time 6 h (OD_600_). The percentage of *P*. *larvae* growth was calculated as follows: $\mathrm{growth} \% =\tfrac{{{\rm{growth}}}_{(\mathrm{larval}\mathrm{food})}-{\mathrm{growth}}_{({\rm{PBS}})}}{{{\rm{growth}}}_{({\rm{PBS}})}}\times 100$. Percentage of *P*. *larvae* growth in the presence of larval food relative to untreated controls (phosphate buffer only) was transformed using arcsine square root transformation $(y=arcsine(\surd \mathrm{growth} \% ))$ and compared among treatment groups by ANOVA with colony as a random factor and treatment as a fixed effect. R version 2.15 was used for analysis, α = 0.05. After transformation, the data fitted a normal distribution and the residuals were equally distributed.

Unchallenged colonies did not show AFB clinical signs; therefore, AFB infection level was compared only between the treatments with propolis + *P*. *larvae* and no propolis + *P*. *larvae*. An overall AFB severity score for each colony by each month (i.e., August, September and October) was obtained by calculating the median of the individual comb scores. AFB severity scores were compared between challenged colonies with and without a propolis envelope using Mann-Whitney U test. R version 2.15 was used for analysis, α = 0.05.

## Electronic supplementary material

Supplemental material

Supplementary figure S1
