# Supplementary material for: Propolis envelope in Apis mellifera colonies supports honey bees against the pathogen, Paenibacillus larvae
Source: Sci Rep. 2017 Sep 12;7:11429. doi: 10.1038/s41598-017-11689-w (PMC5595881; doi:10.1038/s41598-017-11689-w)
Supplement: Supplementary file 1 — Supplemental material [file 41598_2017_11689_MOESM1_ESM.doc]

**Supplemental material**

**Manuscript title:** Propolis envelope in *Apis mellifera* colonies supports honey bees against the pathogen, *Paenibacillus larvae*

Title: 107 characters with space

**Authors:** Renata S. Borba^a^* and Marla Spivak^a^

^a^ Department of Entomology, University of Minnesota, 1980 Folwell Ave. Saint Paul, MN 55108

* To whom correspondence should be addressed: Renata Soares Borba, 1 Research Road, Beaverlodge, Alberta, Canada, T0H 0C0. [rsborba@umn.edu](mailto:rsborba@umn.edu)

**Figure**

**
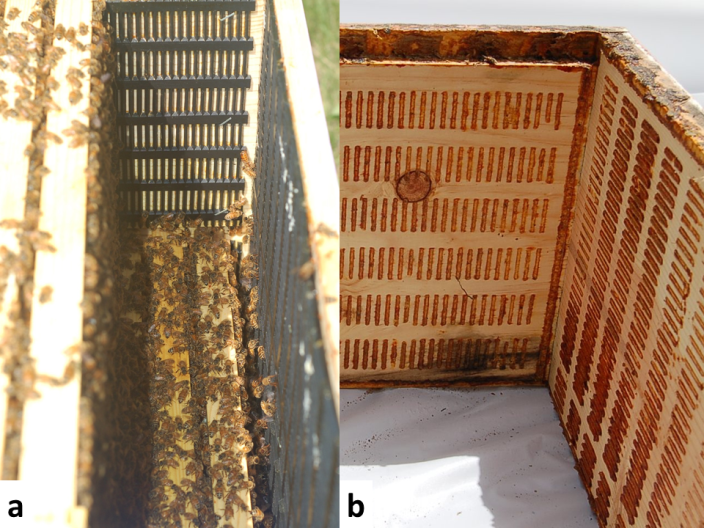
**

**Supplementary figure S1. Propolis envelope treatment bee box. a**) Propolis traps stapled to inside walls of a hive to encourage bees to construct a propolis envelope. b) View of the propolis envelope when traps were removed at the end of the experiment. In each colony, the bees deposited propolis within most of the gaps of each propolis trap (brown lines on the box are the deposited propolis). In a tree cavity, the propolis envelope is contiguous, but bees do not tend to deposit propolis on planed wooden walls in beekeeping equipment, unless lumber is left unfinished.
