# Supplementary figures and images for: Propolis envelope in Apis mellifera colonies supports honey bees against the pathogen, Paenibacillus larvae
Source: Sci Rep. 2017 Sep 12;7:11429. doi: 10.1038/s41598-017-11689-w (PMC5595881; doi:10.1038/s41598-017-11689-w)

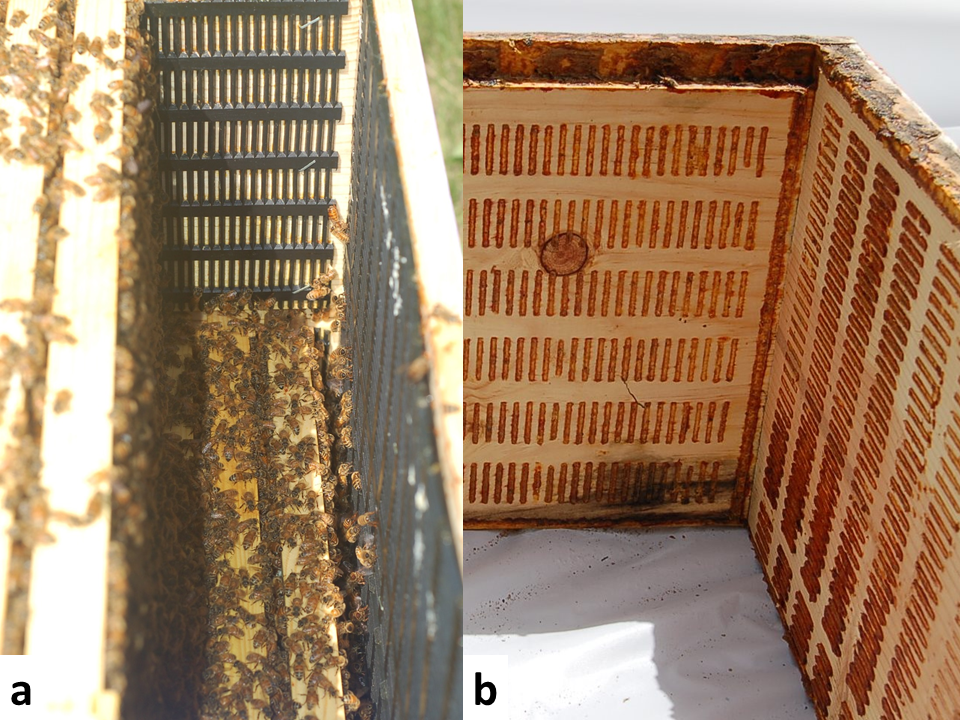

Supplement: Supplementary file 2 — Supplementary figure S1 [file 41598_2017_11689_MOESM2_ESM.tif]
